# EZR promotes pancreatic cancer proliferation and metastasis by activating FAK/AKT signaling pathway

**DOI:** 10.1186/s12935-021-02222-1

**Published:** 2021-10-09

**Authors:** Jian Xu, Wei Zhang

**Affiliations:** 1 Department of Hepatobiliary Surgery 1, Institute of Hepatobiliary-Pancreatic-Intestinal Diseases, Affiliated Hospital of North Sichuan Medical College, No. 1 Maoyuan nan Road, Shunqing District, Nanchang, 637000 China; 2 Department of Nuclear Medicine, Affiliated Hospital of North Sichuan Medical College, Nanchang, 637000 China

**Keywords:** EZR, Pancreatic cancer, Proliferation, Metastasis, FAK/AKT

## Abstract

**Background:**

As a member of the ERM (ezrin-radixin-moesin) protein family, EZR has been recognized as a regulator of adhesion signal pathways by researchers. Moreover, EZR was thought to play irreplaceable roles in invasion and metastasis of versatile cancers. In this study, we managed to undermine the effect of EZR on proliferation and metastasis in pancreatic cancer (PC).

**Methods:**

To analyze the impact of EZR expression on overall survival and free diseases survival of PC patients, we screened abnormally expressed EZR in PC using the Gene Expression Omnibus database (GEO database) and The Cancer Genome Atlas (TCGA) database. Following, Gene Ontology (GO)-based functional analysis and Gene set enrichment analysis (GSEA) was performed to predicate the possible biological processes in which EZR were involved. The clinicopathological characteristics and prognosis of PC patients were analyzed according to clinical data. Further, immunohistochemistry, western blotting and real time PCR analysis were conducted to analyze the expression level of EZR in PC and paired paracancerous tissues. The effect of EZR on proliferation of PC cell lines were detected by Cell Counting Kit-8 assay, and meanwhile, Transwell assay was performed to detect the effect of EZR on invasion and migration of PC cell.

**Result:**

EZR exhibited higher expression level in pancreatic cancer tissues and cell than paracancerous tissues and cell, and its expression level was positively correlated with poor overall survival and diseases-free survival in PC patients. CCK8 assay indicated that EZR facilitated the proliferation of PC cells, meanwhile, Transwell assay showed that EZR promoted the migration and invasion of PC cells. The GO analysis predicated that EZR was involved in biological processes including cell adhesion, ameboidal-type cell migration, cell junction assembly. Through GSEA analysis, pancreatic cancer pathway, and the adhesion junction pathway were screened as the mostly enriched pathways in EZR-regulated pathological process. The inhibition of EZR suppressed proliferation and migration of PC cells. Western blot experiment revealed a positive correlation between EZR and FAK, the proliferation invasion and migration ability of PC cells were significantly decreased after knockdown of EZR.

**Conclusion:**

Our finding revealed EZR accelerated the progression of PC via FAK/AKT signaling pathway.

**Supplementary Information:**

The online version contains supplementary material available at 10.1186/s12935-021-02222-1.

## Introduction

Pancreatic cancer (PC) is generally acknowledged as one of the most malignant cancer which is difficult to diagnose and cure [1]. Despite the technology in diagnosis instrument and treatment for PC have improved, the average 5-year survival rate of PC patients remained lower than 6–8% [2, 3]. Surgery is recognized as the main treatment for pancreatic cancer, while 70–80% of patients have no surgical opportunity when they were diagnosed, moreover, the recurrence rate of PC patients undergone resection is extremely high due to metastasis of tumor [4, 5]. Though the growth of a certain number of PC patients’ survival has witnessed the potential of targeted biological therapy to effectively improve prognosis, it still exists huge obstacles for the prognosis of PC since early local tumor invasion, metastasis and multi-drug resistance. They are ranking the primary cause of low survival rate in PC [6, 7]. Recently, researches on molecular diagnosis have risen more and more recognition, it is also necessary to explore the molecular mechanism to improve the prognosis of patients with PC [8–10]. Consequently, it is urgent to recognize novel prognosis biomarkers for early diagnosis and the exploration of new treatment.

As a member of the ezrin-radixin-moesin (ERM) cytoskeletal proteins, Ezrin (EZR), participates in biological processes including adhesion, migration, cytokinesis, and formation of surface structures [11, 12]. Locating at the top of cell surface, EZR interacts directly with adhesion-related proteins such as CD43, CD44, CD95, ICAM-1, -2, -3 and etc. to maintain the polarity of epithelial cells [13–15]. In recent years, researchers have confirmed that EZR is involved in cellular interaction by adopting adhesion molecules such as Rac1/RhoA and eventually regulate the invasion and metastasis of cancers, and PI3K/Akt signal pathways [16, 17]. Mounting evidence have proved that the expression level of EZR was significantly higher in tumor tissues compared with normal tissues. Zhang et al. [18] demonstrated that the expression level of EZR in breast tumor tissues was significantly higher compared to normal tissues. Moreover, High expression of EZR was accompanied with poor overall survival (OS) in breast cancer patients. The expression of EZR was significantly higher than paired normal tissues in colorectal cancer, meanwhile, abnormal expressed EZR was correlated with higher Dukes stage, lower degree of differentiation and lymph node metastasis [19].

To our knowledge, the function of EZR has not been reported in PC. This research aimed to investigate the role of EZR in the progression of PC and explore the underlying molecular mechanisms of EZR in tumorigenesis, EZR was found to be abnormally expressed in PC by the Gene Expression Omnibus database (GEO database) and The Cancer Genome Atlas (TCGA) database analysis. In addition, EZR functioned as tumor promoter in facilitating cell proliferation, migration and invasion in PC cells. Moreover, we also explored the modulatory mechanism of EZR in the development of PC as it might serve as a therapeutic marker for PC treatment.

## Materials and methods

### EZR expression analysis

We searched the GEO and TCGA databases to download pancreatic cancer data and compare the human pancreatic cancer tumor samples with normal samples. GEO datasets (GSE10144845 and GSE107610) and TCGA dataset were utilized to measure the gene expression of EZR, then we obtained the heat map using RStudio software. GEPIA (http://gepia.cancer-pku.cn/) was used to analyze RNA sequencing expression data from samples (contains tumor and normal) of TCGA. The screening conditions contains: (1) selecting datasets: pancreatic cancer; (2) gene: *EZR*; (3) expression DIY: boxplot; and (4) matched normal data. Survival analysis was also performed using online web server GEPIA (http://gepia.cancer-pku.cn/).

### Construction of protein–protein interaction network

Protein–protein interaction (PPI) network of EZR was established through Search Tool for the Retrieval of Interacting Genes/Proteins (STRING) database. Correlation genes of EZR were screened using Gene Ontology (GO)-based functional analysis. p < 0.05 was set as the cut-off criterion.

### Gene set enrichment analysis

The Gene Set Enrichment Analysis (GSEA) software (version 3.0) and Java software were used to analysis EZR. The “uniq.symbol.txt” data set was downloaded, the high-to-low grouped expression profile data was enriched and analyzed by default weighted enrichment statistics.

### Patient data and tissue sample

The PC tissues and paired paracancer tissue were obtained from 26 patients who underwent surgical treatment between 2018 and 2020 at the Affiliated Hospital of North Sichuan Medical college, Nanchong, China. All tissue were confirmed by histopathological examination and snap frozen in liquid nitrogen immediately after operation and then stored at − 80 °C. None of the patients have received any local or systemic therapy before surgery. All patients registered were informed and consent. All experiments were permitted by the research ethic committee of North Sichuan Medical college.

### Immunohistochemical (IHC)

PC and paracancer tissues were collected and fixed in 10% formalin. After a 15-min antigen retrieval protocol in 0.01 M sodium citrate buffer (pH 6.0) at room temperature, the sections were blocked in 0.3% H_2_O_2_ and incubated for 1 h with Rb-EZR [20]. Then, the sections were labeled with DAB and stained with hematoxylin. The sections were baked again prior to imaging, relevant analysis using a microscopic image analysis system (DS-Ri2, NIKON). The results were evaluated as 0, negative; 1, weakly positive; 2, moderately positive; or 3, strongly positive. We used the accurate formula to calculated the staining index: staining index = staining intensity × percentage of positive cells. Low expression was defined as a staining index < 5 [21].

### Cell culture

PANC-1 and MIA PaCa-2 cell lines were purchased from ATCC (Manassas, USA), Human normal pancreatic ductal cells (HPDE), AsPC-1 and BxPC-3 cell lines were purchased from CASCB (Shanghai, China). All cells were grown in DMEM (Gibico, Carlsbad, CA, USA) medium supplemented with 10% fetal bovine serum (Gibico, Carlsbad, CA, USA) and 1% penicillin/streptomycin (Beijing Solarbio Science, Beijing, China) at 5% CO_2_ and 37 °C.

### Transfection

EZR siRNAs and scrambled negative control (NC) siRNAs were purchased from RiboBio, Guangzhou, China. The cells were seeded and cultured in six-well plate with density of 3 × 10^5^/well overnight. Then, cells were transfected with siRNAs or negative control at a final concentration of 50 nM using Lipofectamin 2000 reagent (Invitrogen, Carlsbad, USA) [22].

### Quantitative real-time PCR (QRT-PCR) analysis

Total RNA was extracted from all cells, a TRIzol kit was used (Invitrogen, Carlsbad, CA, USA). Complementary DNA (cDNA) was used 2 μg of the total RNA according to the instructions of the reverse transcriptase kit (Takara Bio, Inc., Dalian, China) in a LifePro Thermal Cycler (Hangzhou Bioer Technology Co. Ltd., Hangzhou, China) [23]. SYBR Premix Ex Taq (Takara, Japan) was used for Quantitative real-time PCR assay on the CFX Connect Real-Time System (Bio-Rad, USA). β-actin was used as the an internal reference gene for normalization. The forward and reverse primers for EZR were 5′-ACCAATCAATGTCCGAGTTACC-3′ and 5′-GCCGATAGTCTTTACCACCTGA-3′, respectively. The primers for GAPDH were 5′-AACGGATTTGGTCGTATTGG-3′ and 5′-TTGATTTTGGAGGGATCTCG-3′, respectively. Relative expression levels of genes were calculated by using the comparative cycle threshold (Ct) (2^−ΔΔ^Ct) method and then converted to fold-changes.

### Cell proliferation and metastasis assay

A cell proliferation assay was implemented with a CCK-8 assay kit (Dojindo Laboratories Co. Ltd, Kumamoto, Japan). Briefly, cells (5 × 10^3^ cells/well) were seeded into 96-well plates with 100ul per well of DMEM culture medium supplemented with 10% FBS and cultured at 37 °C and 5% CO2 atmosphere. Each sample has six replicates. The medium was replaced by 100 μl fresh culture medium, and 10 μl CCK-8 solution was added to each well for different periods of time (0, 24, 48, 72 and 96 h). Each well was measured spectrophotometrically at 450 nm using a Quant ELISA Reader (BioTek Instruments, USA) after 2 h of incubation. For transwell assay, the chamber inserts were precoated with Matrigel (1:8 ratio in DMEM), 2 × 10^4^ cells in 200 μl of serum-free medium were seeded into the upper chamber of each well. The lower chambers were filled with 800 µl DMEM supplemented with 20% FBS, after 24 h, PC Cells were fixed, stained with 4% paraformaldehyde and 0.4% crystal violet, respectively. 50 μl Matrigel was pre-coated in the upper chamber for migration assay, and using the similar procedure to invasion assay. Following, 10 fields in each chamber were observed to evaluate the number of invasion cells of corresponding group, average of those fields were utilized as the final result. All experiments were performed in quintuplicate and repeated once.

### Colony formation assay

For the colony formation assay, Cells (500 cells/well) were seeded into 6-well plates maintained in DMEM media containing 10% FBS for 2 weeks replacing the medium every 4 days. After fixation in 4% paraformaldehyde for 10 min, cells were stained with 1% crystal violet. Colonies with diameters greater than 100 μm were counted. Each sample was assessed in triplicate.

### Co-immunoprecipitation and Western blot analysis

Co-immunoprecipitation (IP): PC cells were harvested, then resuspended in RIPA for 15 min. Cell lysate was centrifuged at 14,000 rpm for 15 min. The supernatant incubated with primary antibody (or IgG) and Pierce Protein G Agarose, The beads were washed and resuspended with sample, then heated at 100 °C for 5 min. quantified total protein with BCA method. Equal amount of lysate was separated and then transferred to the PVDF membrane. The membrane with target protein was incubated with the primary antibody at 4 °C and the second antibody was incubated for 1 h at room temperature. For Western blotting, total protein was extracted using RIPA with a Protease Inhibitor Cocktail. Then, the protein samples were transferred onto a PVDF membrane, which was followed by blocked with 5% fat-free milk at room temperature for 2 h, and an incubation overnight at 4 °C in a 1:500 dilution of primary antibodies. The membranes were washed three times with Tris-buffered saline containing Tween-20 (TBST), and then the membrane was incubated with HRP-conjugated rabbit or mouse secondary antibodies for 2 h. The intensity of the protein band was densitometrically quantified using Image J software (version 1.50i).

### Statistical analysis

All statistical analyses were performed with the SPSS 22.0 statistical software package (IBM, Chicago, USA) and GraphPad Prism 6.0 software (GraphPad software, USA). Two-sided p values were calculated, and a threshold of p < 0.05 was considered statistically significant. The results are expressed as mean ± SD. Statistical significance was assigned at *p < 0.05 or **p < 0.01. All experiments were carried out at least three times, in triplicate samples.

## Results

### Bioinformatics analysis of gene expression profiles: EZR is significantly up-regulated in PC

**Fig. 1:**
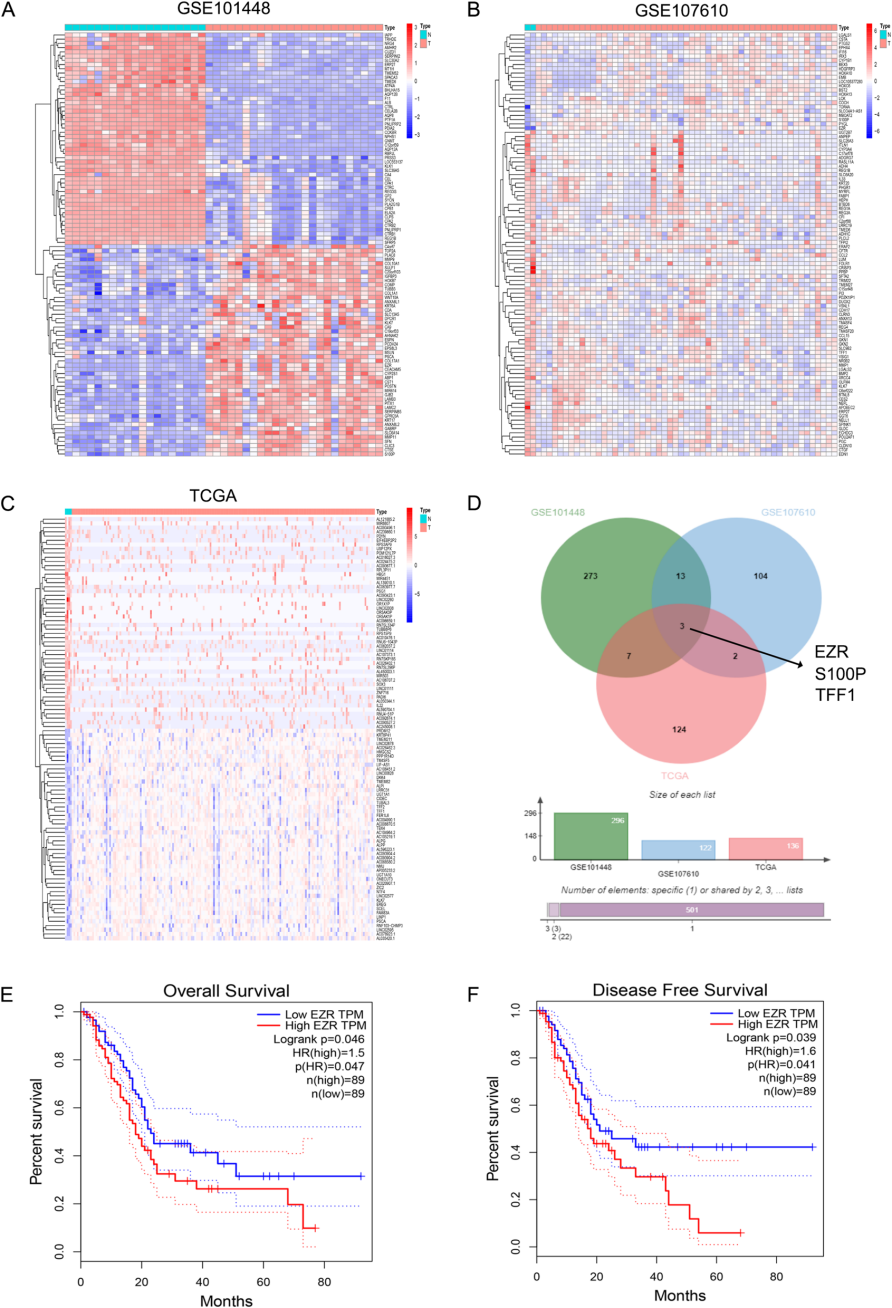
Bioinformatics analysis of gene expression profiles to find EZR. **A**, **B** Heat map of the differentially expressed genes in the microarray datasets from GEO (GSE101448 and GSE15471). **C** Heat map of the differentially expressed genes in the microarray dataset from TCGA. **D** Venn diagram of the differentially expressed genes in the three microarray datasets. **E**, **F** Kaplan–Meier survival analysis revealed that PC patients with high expression of EZR had a poor diagnosis in overall survival rate (OS) and disease free survival (DFS) according to GEPIA (http://gepia.cancer-pku.cn/), the median expression level of EZR was set as cutoff for patients classification

The GEO and TCGA databases were utilized to measure the expression of different genes in PC and normal pancreatic tissues, the differentially expressed genes were shown in Fig. 1A–C, Venn diagram of the differentially expressed genes in the three microarray datasets was shown in Fig. 1D, we obtained three target genes S100P, EZR and TFF1, then Kaplan–Meier survival analysis demonstrated that PC patients with high expression of EZR was positively correlated with diagnosis in overall survival rate (OS) and disease free survival (DFS) according to GEPIA database (Fig. 1E, F). In contrast, the other two genes had no effect on OS or DFS of PC patients (Additional file 1: Figure S1). These results demonstrated that EZR was highly expressed in PC, and was also related to survival rates in PC patients.

### EZR is highly expressed in PC tissues and cell lines

**Fig. 2:**
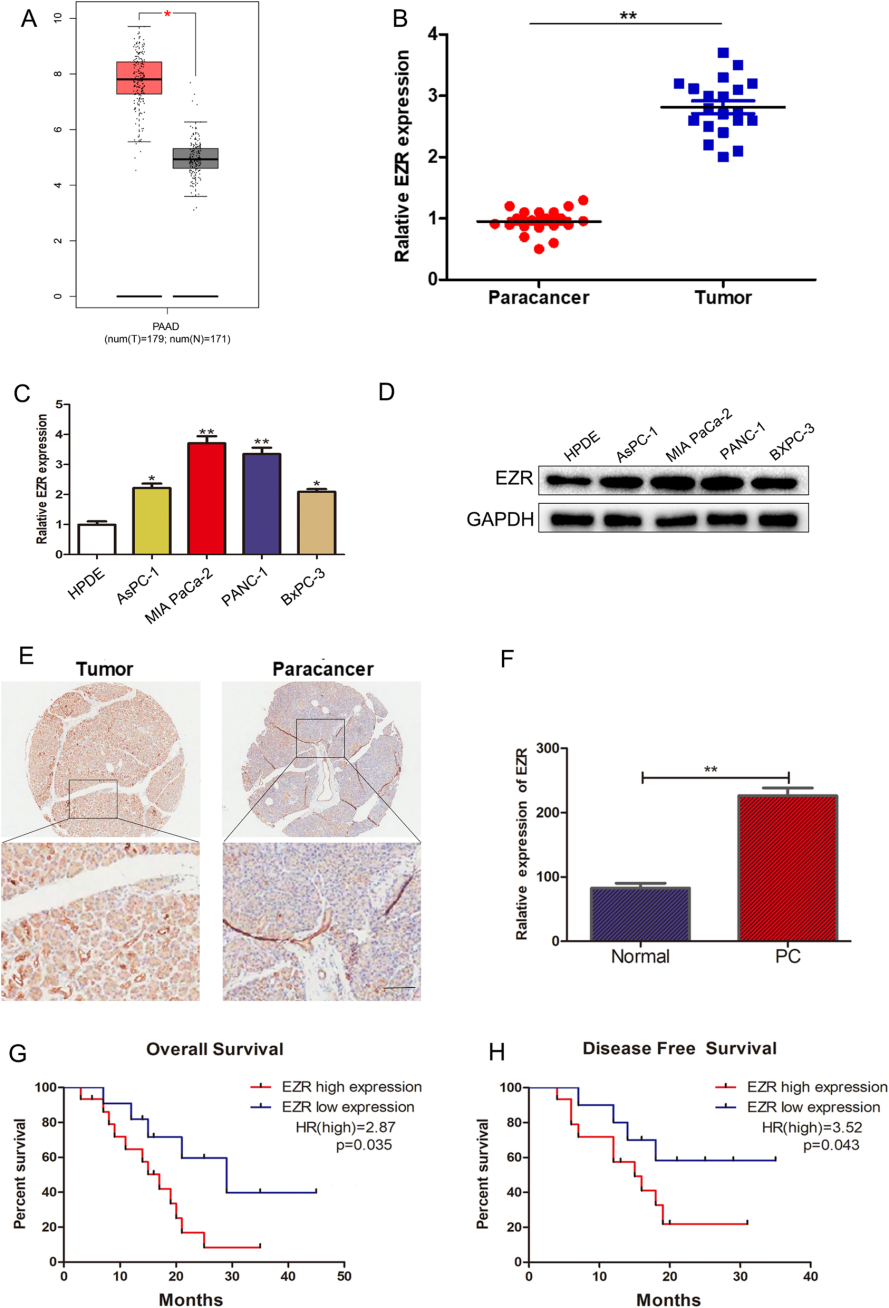
Significantly upregulated EZR expression in PC tissues and PC cell lines, PC patients with high expression of EZR had a poor diagnosis. **A** GEPIA data of EZR expression in PC tissues and normal pancreatic tissues (*p < 0.05). **B** 26 paired cancer tissues and paracancer tissues were collected from patients, EZR was aberrantly upregulated in PC tissues compared with paracancer tissues (**p < 0.01). **C**, **D** RT-qPCR and WB assessed EZR expression in normal pancreatic cell line (HPDE) and PC cell lines (AsPC-1, PANC-1, BXPC-3, and MIA PaCa-2). **E** Immunohistochemical detection of EZR expression in PC tissues compared with paracancer tissues. **F** The difference of histochemistry score between PC tissues and paracancer tissues. **G**, **H** Kaplan–Meier survival analysis verified high expression of EZR had a poor diagnosis in 26 PC samples. *p < 0.05, **p < 0.01, ***p < 0.001

EZR, which has been reported to be overexpressed in human cancers, such as breast cancer [18] and colorectal cancer [19], is significantly upregulated in PC tissues. According to GEPIA (http://gepia.cancer-pku.cn/) database, EZR was highly expressed in PC tissues (Fig. 2A, p < 0.05). Moreover, we verified the upregulation of EZR in PC samples compared to paracancer samples in 26 PC specimens (Fig. 2B, p < 0.01). Then, the expression level of EZR in the PC cell lines and normal pancreas epithelial cell line (HPDE) were compared using Quantitative RT-PCR and Western blot experiment. The results showed that the expression of EZR was significantly higher in the PC cell lines compared to HPDE (Fig. 2C, D), especially in PANC-1 and MIA PaCa-2 cell lines. Analysis of 26 PC samples revealed the correlation between the EZR expression and clinicopathological features of PC: high expression of EZR was significantly correlated with lymph node metastasis (p = 0.021) and TNM stage (p = 0.045) (Table 1). Data of immunohistochemical further confirmed the high positivity of EZR in PC tissues (Fig. 2E, F). Kaplan–Meier survival analysis verified that high expression of EZR was accompanied by poor diagnosis in 26 PC samples (Fig. 2G, H; p < 0.05). Taking together, these data indicated that EZR was significantly increased in PC tissues and might serve as a novel biomarker for the diagnosis and prognosis of PC patients.

**Table 1:** General clinicopathological characteristics of patients

Clinical epidemiology and clinicopathologic feature	N	EZR		*P value*
		Low expression	High expression	
All cases	26	11	15	
Age				0.453
≤ 60	12	4	8	
> 60	14	7	7	
Gender				0.689
Male	16	6	10	
Female	10	5	5	
Diameter of tumor				0.701
≤ 3	15	7	8	
> 3	11	4	7	
Tumor differentiation				0.692
Well/moderate	14	5	9	
Poor	12	6	6	
Pathological T				0.428
T1/T2	16	8	8	
T3/T4	10	3	7	
Lymph node metastasis				0.021
N0 (negative)	14	9	5	
N1 (positive)	12	2	10	
TNM stage				0.045
I/II	14	8	4	
III/IV	13	3	11	
Vessel invasion				0.109
Negative	16	9	7	
Positive	10	2	8	

Low/high by the sample median, used Fisher’s exact test

^*^p < 0.05 was considered to be statistically significant

### EZR promotes PC cells proliferation, metastasis, invasion and migration

**Fig. 3:**
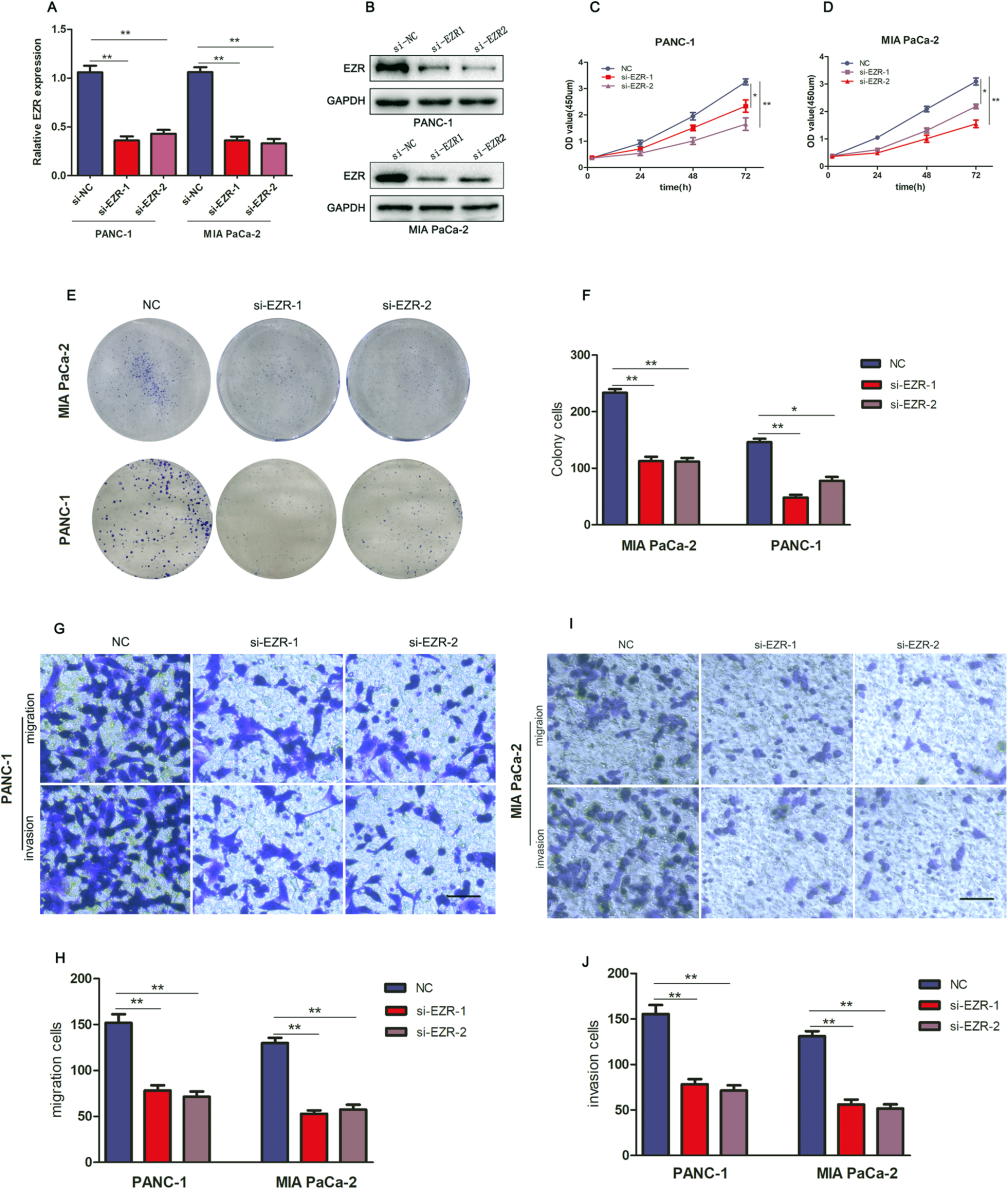
EZR promotes PC cells proliferation, invasion and migration. **A** RT-qPCR identified the expression of EZR was lower in si-EZR1/2-transfected cells than corresponding negative control group cells. **B**, **C** CCK-8 assay showed that down-regulation of EZR significantly reduced cell viability in PC cell lines. **D** si-EZR1/2 of MIA PaCa-2 and PANC-1 cells suppressed the colony formation ability respectively. **F**, **G** the cell migratory and invasive capacity of si-EZR1/2(MIA PaCa-2 and PANC-1 cells) were significantly repressed. The images of data quantification of **E** colony formation, **H** transwell migration and **I** invasion assays. *p < 0.05, **p < 0.01, ***p < 0.001

To identify the effect of EZR on PC cells, we measured the cellular proliferation, invasion and migration capabilities of MIA PaCa-2 and PANC-1 cells transfected with negative control vector (NC) or EZR silencing sequence (si-EZR-1 or si-EZR-2) which knocked down EZR expression, the expression of EZR was detected by RT-qPCR and Western blot experiment (Fig. 3A, B). CCK-8 assay showed that knocking down of EZR expression significantly reduced cell viability in PC cells (Fig. 3C, D); compared with the control groups, the knockdown of EZR (si-EZR-1 and si-EZR-2) suppressed the cell viability and colony formation ability in both MIA PaCa-2 and PANC-1 cells (Fig. 3E). On the other hand, the cell migratory and invasive capacity of MIA PaCa-2 and PANC-1 cells transfected with si-EZR1/2 were significantly repressed compared with the control group (NC) (Fig. 3F–I). Overall, the cell functional data manifested that EZR promoted proliferation, invasion and migration capacity of PC cell.

### Bioinformatics analysis revealed the EZR-involved biological function and enrich relevant genes

**Fig. 4:**
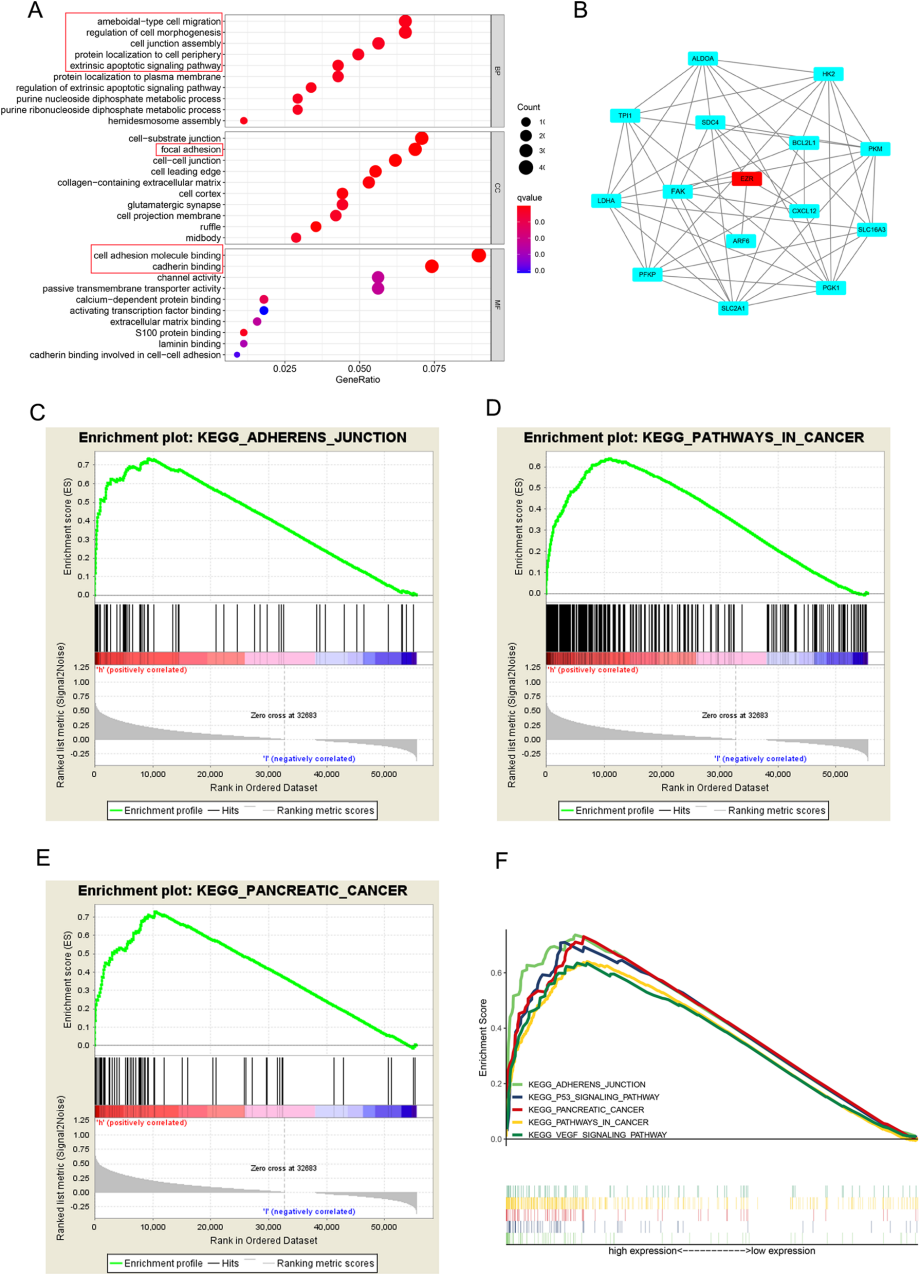
Bioinformatics analysis predicted the biological function of EZR. **A** GO enriched the cellular component organization (CC), biological process (BP) and molecular function (MF). The results showed EZR was involved in cell adhesion molecule binding, focal adhesion, ameboidal-type cell migration, regulation of cell morphogenesis and etc. **B** The protein–protein interaction (PPI) network of EZR was constructed using STRING. **C**–**E** GSEA showed pathways involved in the EZR enrichment analysis: adherens junction, pathways in cancers and especially in pancreatic cancer were selected as candidates. The data was analyzed from TCGA-PAAD database and demonstrated by Cytoscape. **F** Multi GSEA enrichment showed the higher expression of EZR could strongly active the five signaling pathways

EZR and relevant genes were mainly screened by GO analysis in three aspects including the cellular component organization (CC), biological process (BP) and molecular function (MF). The results showed that EZR was correlated with cell adhesion molecule binding, focal adhesion, ameboidal-type cell migration, regulation of cell morphogenesis, etc. (Fig. 4A). We used the search tool for the retrieval of interacting genes (STRING) (http://string-db.org/cgi/input.pl) to construct the protein–protein network (PPI) of EZR, a total of 14 proteins were predicted directly interacted with EZR, among which, FAK was found to be positively correlated with EZR in PC (Fig. 4B). Taking previous studies into Consideration, the FAK/AKT signaling pathway was reported to correlate with cancer cell proliferation and metastasis [22], we performed GSEA in TCGA-PAAD to analyse the biological process influenced by EZR. As our results depicted, EZR were related to pathological pathways including adherens junction pathways in pancreatic cancer, p53 signaling pathway and VECF signaling pathway. Our finding demonstrated that EZR might play an important role in the progress of PC (Fig. 4C–F).

### The effect of EZR on the FAK/AKT signaling pathway

**Fig. 5:**
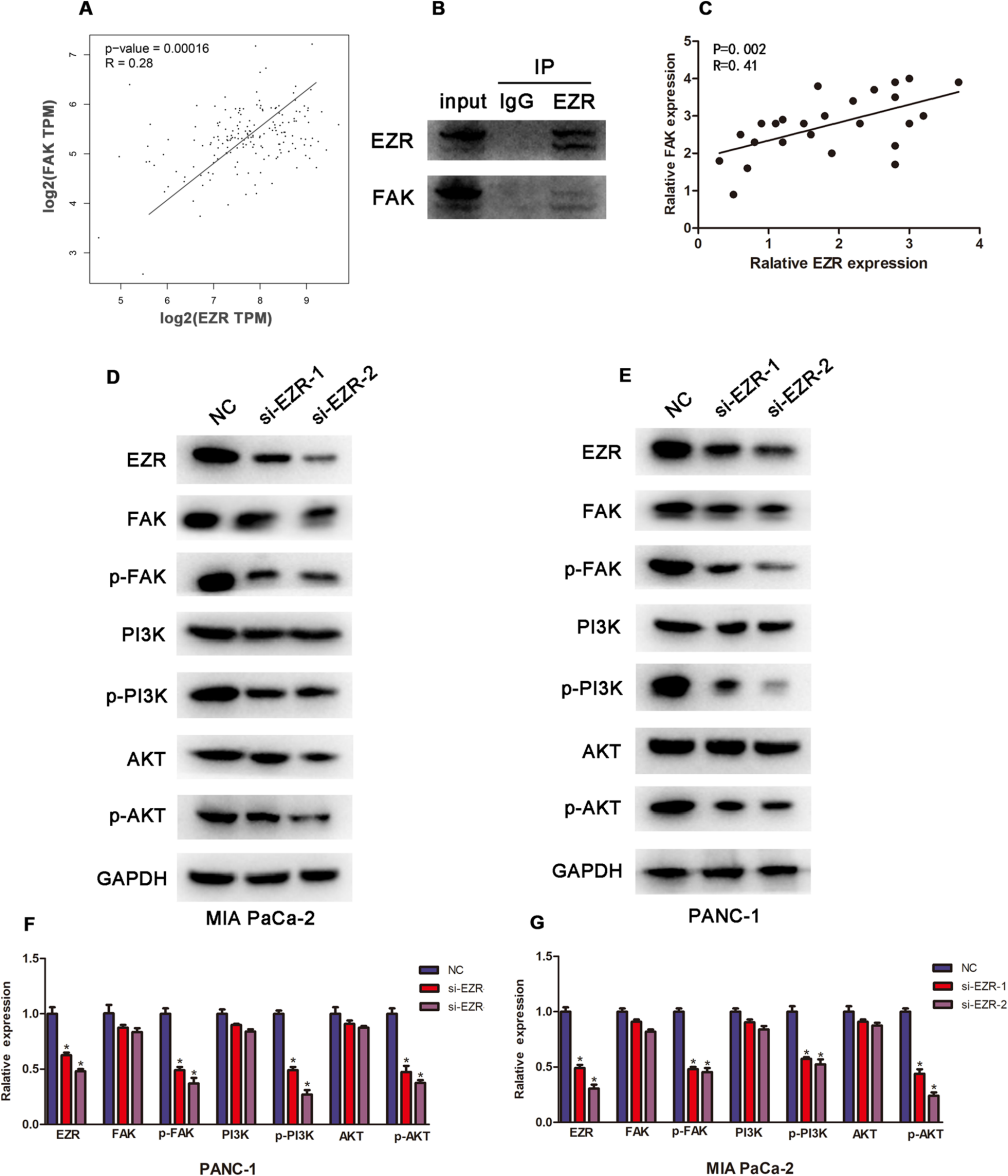
The effect of EZR on the FAK/AKT signaling pathway. **A** FAK was to predicted to have a positive correlation with EZR in PC using GEPIA (http://gepia.cancer-pku.cn/) (*p*  = 0.00016). **B**, **C** Immunoprecipitation (IP) and RT-qPCR comfirmed the positive correlation between EZR and FAK. **D**–**G** Western Blot showed that si-EZR could inhibit the expression of p-FAK, p-PI3K and p-AKT, but could not decrease the total expression level of FAK, PI3K and AKT in MIA PaCa-2 (**D**, **F**) and PANC-1cell lines (**E**, **G**). *p < 0.05, **p < 0.01, ***p < 0.001

As FAK was found to have a significantly positive correlation with EZR in PC by using GEPIA (http://gepia.cancer-pku.cn/) (Fig. 5A), we further confirmed the interaction between EZR and FAK through Immunoprecipitation (IP) and RT-PCR experiment (Fig. 5B, C). The FAK/AKT signaling pathway had been reported to correlate with cell proliferation and metastasis [22, 23], to verify whether the biological function of EZR was achieved through FAK/AKT pathway, the expression levels of EZR and associated proteins of FAK/AKT signaling pathway were analyzed in EZR knockdown PANC-1 (Fig. 5D, F) and MIA PaCa-2 cell lines (Fig. 5E, G) via Western Blot experiment. The results showed that si-EZR could inhibit the expression of p-FAK, p-PI3K and p-AKT, but could not decrease the total expression level of FAK, PI3K and AKT.

### EZR promoted the progression of PC via up-regulating FAK

**Fig. 6:**
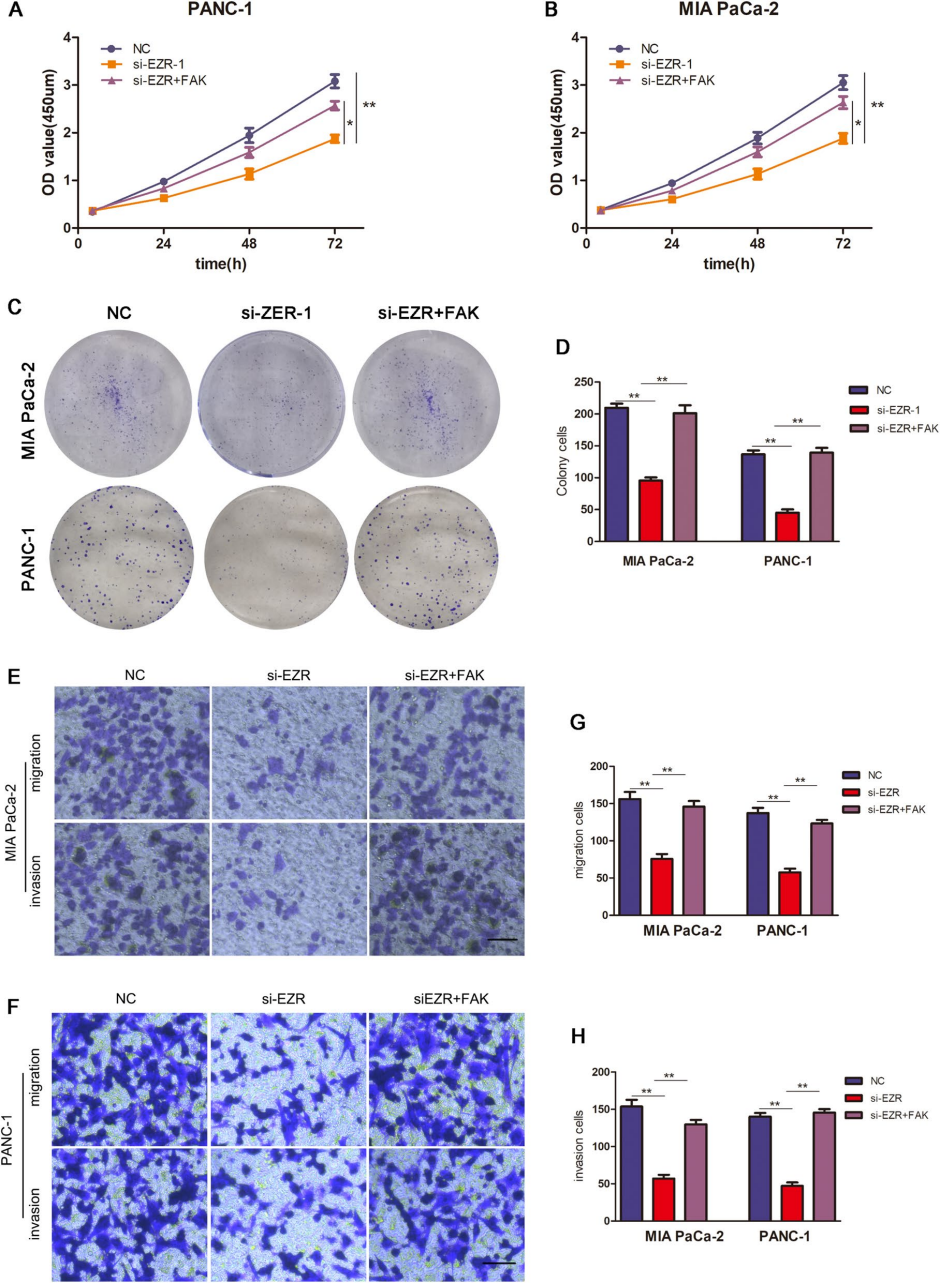
EZR promoted the progression of PC via up-regulating FAK. **A**, **B** CCK-8 assay showed the proliferation ability of EZR co-transfected with si-EZR-1 and FAK. **C** colony assay, PC cells viability and colony formation ability was neutralized after the co-transfection of FAK. **E**, **F** The cell migratory and invasive capacity of MIA PaCa-2 and PANC-1 cells repressed by si-EZR-1 were reversed. The images of data quantification of colony formation, transwell migration and invasion assays were displayed in **D**, **G**, **H** respectively. *p < 0.05, **p < 0.01, ***p < 0.001

**Fig. 7:**
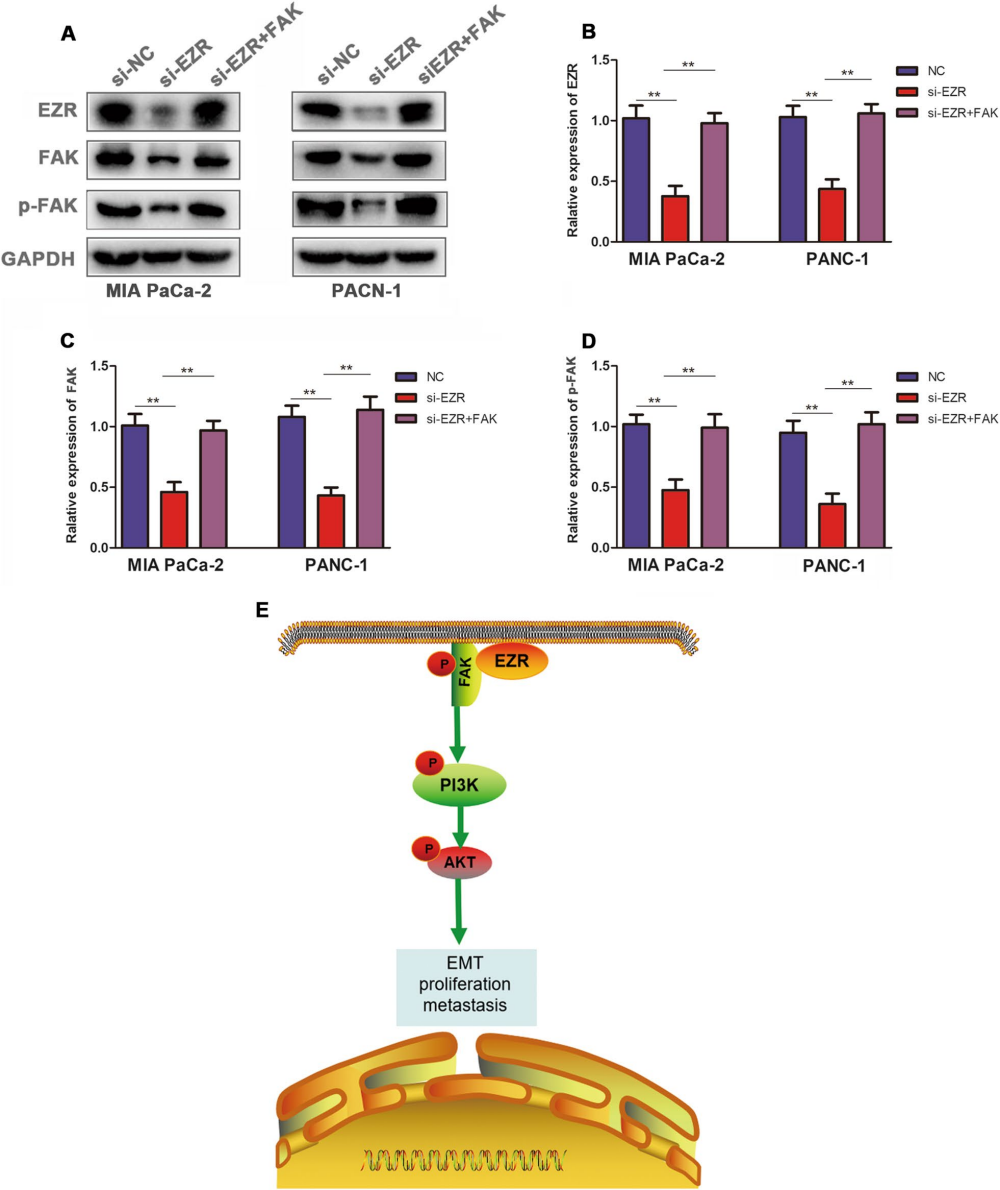
EZR promoted the progression of PC and schematic illustration of EZR-FAK/AKT signaling pathway. **A** The expression of FAK and p-FAK were reversed by co-transfected FAK in MIA PaCa-2 and PANC-1 cells. **B**–**D** The images of data quantification of EZR, FAK and p-FAK in western blot experiment. **E** Schematic illustration of EZR-FAK/AKT signaling pathway. *p < 0.05, **p < 0.01, ***p < 0.001

To confirm the interaction between EZR and FAK, we performed rescue assays to validate whether EZR modulated FAK to influence the proliferation, invasion and migration of PC. As data showed, co-transfection of FAK and si-ERZ-1 resulted in enhancing expression of FAK in PC cells. It was proved that the falling trend of CCK-8 assay induced by si-EZR depletion was then recovered after the co-transfection of FAK (Fig. 6A, B). Compared with the si-EZR group of colony assay, PC cells viability and colony formation ability were suppressed by si-EZR, but the descending tendency was neutralized after the co-transfection of FAK (Fig. 6C, D). Besides, the cell migratory and invasive capacity of MIA PaCa-2 and PANC-1 cells repressed by si-EZR were reversed by the co-transfected FAK respectively (Fig. 6E, G and Fig. 6F, H). At last, the results of Western Blot confirmed that EZR promoted the progression of PC via up-regulating FAK (Fig. 7A–D).

## Discussion

Pancreatic cancer (PC) is a malignant tumor with extremely high mortality and low morbidity which has become one of the leading causes of cancer-related deaths [24–26]. Currently, the prognosis of PC patients is poor, and the 5-year survival rate of PC patients is still low, the current 5-year overall survival (OS) rate of PC patients is estimated to be approximately 6% [9, 25], and the lacking of effective prognostic biomarkers makes it difficult to diagnosis and intervene in the early stages of PC. With the development of gene sequencing technology, many molecular markers have been screened to provided important auxiliary means for prediction of cancer prognosis [27–29], but there are few factors to be identified significantly relating to pancreatic cancer. Therefore, it’s of great importance to understand the molecular mechanisms of PC carcinogenesis and finding reliable PC diagnostic and therapeutic targets.

EZR was frequently highly expressed in severe cancers compared to paired paracancer tissues, such as esophageal squamous carcinoma (ESCC) [30, 31], malignant melanoma [32], ovarian carcinoma [33] and breast cancer [18, 34], etc. EZR was able to remodel cytoskeleton of cancer cell or infiltrate the paracancerous tissues through forming complex with calyx glycoprotein and activating calyx glycoprotein [35, 36]. The locations of EZR were different between cancer cells and normal cells, EZR was located in cell membrane and cytoplasm in cancers cells, while in normal cells it was enriched in actin-rich microvilli and pseudopodia [36, 37]. Meanwhile, EZR participated in the regulation of adhesion molecules and signal pathways involved in the migration and invasion cancer cells [35, 38]. Zhang et al. [18] reported the expression level of EZR in breast cancer tissues was significantly upregulated compared to normal breast tissues (*p* < 0.01), high expression of EZR was correlated with poor overall survival (OS) in breast cancer, but high expression of EZR was not correlated with poor disease-free survival (DFS). Lugowska et al. [39] suggested that preoperative chemotherapy could reverse the overexpression of EZR, it could be a useful predictive and prognostic marker in patients with osteosarcoma. Chang et al. [40] certificated the pancreatic ductal adenocarcinoma-derived Small extracellular vesicles sEVs-Ezrin (sEV-EZR) could modulate macrophage polarization, and were correlated with pancreatic ductal adenocarcinoma metastasis. It maybe a potential therapy to inhibit PDAC metastasis by targeting sEV-EZR. Xiaolong Zhang et al. [41] found baicalein significantly decreased EZR tension through downregulating cellular ezrin S‐nitrosylation (SNO) levels in NSCLC cells by using a genetic encoding tension probe, and decreasing ezrin tension inhibited the migration ability of NSCLC cell, The overexpression of EZR was correlated with the invasion capacity in hepatocellular carcinoma cell lines, transfection of antisense oligonucleotides could significantly inhibit invasion capacity of cells [42, 43].

In our study, we have firstly investigated the expression of EZR in pancreatic cancer, our study focused on biological function (proliferation, migration and invasion), and signaling pathway involving EZR through bioinformatics analysis and immunohistochemistry assay. We found EZR was upregulated in pancreatic cancer tissue compared to paracancer tissue by bioinformatics analysis, then we verified the EZR was overexpressed in PC samples by analyzing our PC tissues. RT-qPCR assessed that EZR was overexpressed in PC cell lines (especially in PANC-1 and MIA PaCa-2 cell lines). Knocking down of *EZR* expression significantly suppressed the cell viability and colony formation ability of PC cell. Meanwhile, the cell migratory and invasive capacity were significantly repressed in PC cells treated with si-EZR. These cell functional data manifested that EZR promoted PC cell proliferation, invasion and migration. The descending tendency of cells viability, colony formation ability, migratory and invasive capacity suppressed by si-EZR were reversed by the co-transfected FAK. The results indicated EZR promoted the progression of PC via up-regulating FAK (Fig. 7E).

In all, we showed that EZR promoted pancreatic cancer cell proliferation migration and invasion via up-regulating FAK. EZR can be used as a potential target for the diagnosis and treatment of pancreatic cancer.

## Supplementary Information

**Additional file 1: Figure S1.** Analysis of the relationship between genes(S100P and TFF1) and PC patients overall survival rate(OS) and disease free survival(DFS) on the Kaplan–Meier plotter database according to GEPIA.

## Data Availability

All data generated or analyzed during this study are included in this published article and its additional files.
