# Supplementary figures and images for: EZR promotes pancreatic cancer proliferation and metastasis by activating FAK/AKT signaling pathway
Source: Cancer Cell Int. 2021 Oct 9;21:521. doi: 10.1186/s12935-021-02222-1 (PMC8502343; doi:10.1186/s12935-021-02222-1)

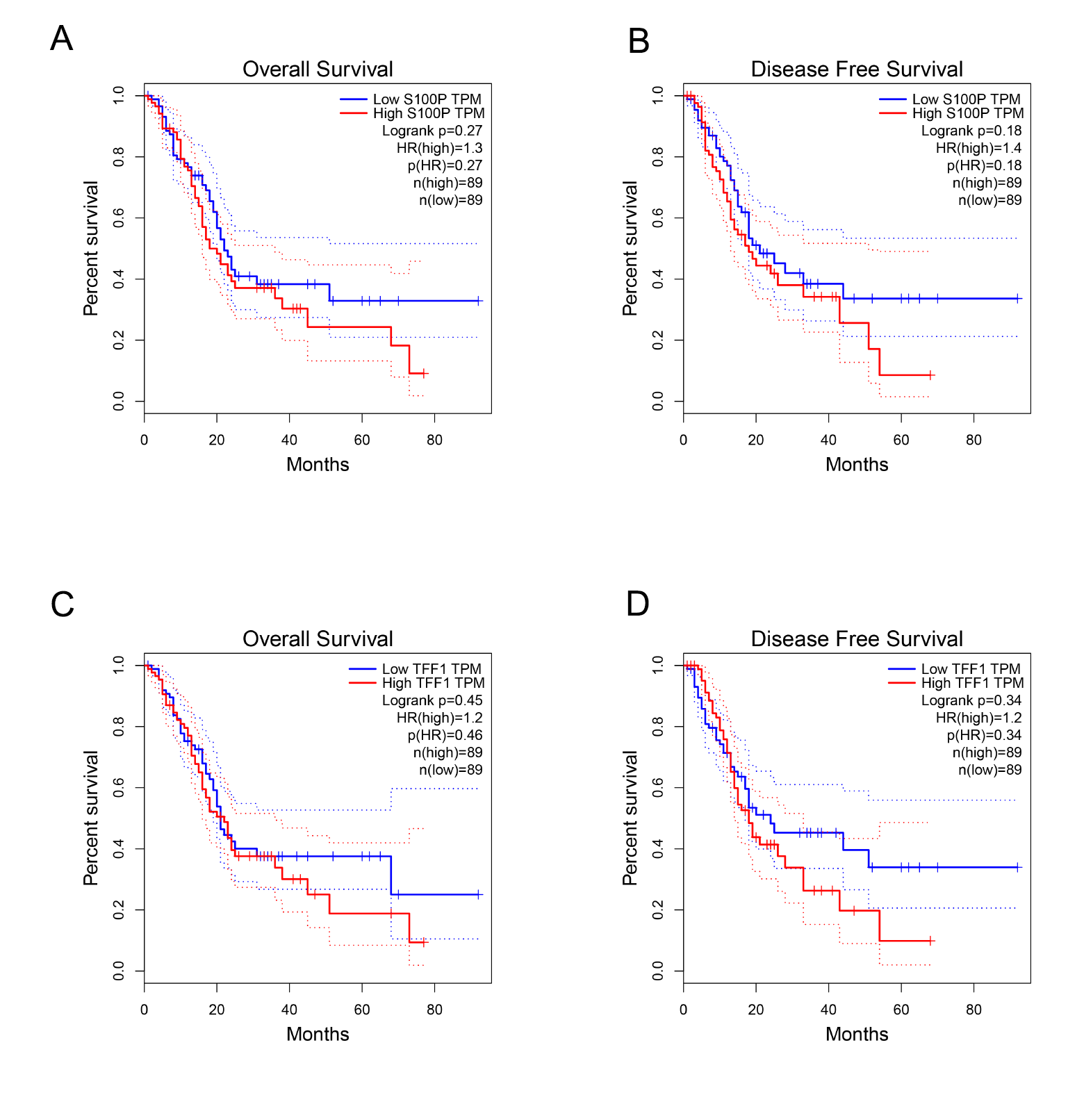

Supplement: Supplementary file 1 — Additional file 1: Figure S1. Analysis of the relationship between genes(S100P and TFF1) and PC patients overall survival rate(OS) and disease free survival(DFS) on the Kaplan–Meier plotter database according to GEPIA. [file 12935_2021_2222_MOESM1_ESM.tif]
